# Mutagenesis of the NaChBac sodium channel discloses a functional role for a conserved S6 asparagine

**DOI:** 10.1007/s00249-017-1246-2

**Published:** 2017-08-20

**Authors:** Andrias O. O’Reilly, Anja Lattrell, Andrew J. Miles, Alexandra B. Klinger, Carla Nau, B. A. Wallace, Angelika Lampert

**Affiliations:** 1 Institute of Physiology and Pathophysiology, Friedrich-Alexander Universität Erlangen-Nürnberg, Universitätsstraße 17, 91054 Erlangen, Germany; 2 School of Natural Sciences and Psychology, Liverpool John Moores University, Liverpool, L3 3AF UK; 3 Department of Anesthesiology, Friedrich-Alexander Universität Erlangen-Nürnberg, Krankenhausstrasse 12, 91054 Erlangen, Germany; 4 Institute of Structural and Molecular Biology, Birkbeck College, University of London, London, WC1E 7HX UK; 5 Institute of Physiology, RWTH Aachen University, Pauwelsstrasse 30, 52074 Aachen, Germany; 6 Department of Anesthesiology and Intensive Care, University of Lübeck, Ratzeburger Allee 160, 23538 Lübeck, Germany

**Keywords:** Ion channel inactivation, Whole-cell patch clamp, Molecular modeling, Circular dichroism spectroscopy, Thermal stability

## Abstract

Asparagine is conserved in the S6 transmembrane segments of all voltage-gated sodium, calcium, and TRP channels identified to date. A broad spectrum of channelopathies including cardiac arrhythmias, epilepsy, muscle diseases, and pain disorders is associated with its mutation. To investigate its effects on sodium channel functional properties, we mutated the simple prokaryotic sodium channel NaChBac. Electrophysiological characterization of the N225D mutant reveals that this conservative substitution shifts the voltage-dependence of inactivation by 25 mV to more hyperpolarized potentials. The mutant also displays greater thermostability, as determined by synchrotron radiation circular dichroism spectroscopy studies of purified channels. Based on our analyses of high-resolution structures of NaChBac homologues, we suggest that the side-chain amine group of asparagine 225 forms one or more hydrogen bonds with different channel elements and that these interactions are important for normal channel function. The N225D mutation eliminates these hydrogen bonds and the structural consequences involve an enhanced channel inactivation.

## Introduction

Members of the six-transmembrane helix (6TM) ion channel family play a role in many diverse physiological processes including sensory perception, action potential propagation, and cell signaling (Hille 2001), whereas their dysfunctional expression or mutation results in a wide range of human neurological and cardiovascular channelopathies (Lehmann-Horn and Jurkat-Rott 1999; Lampert et al. 2010; Kwong and Carr 2015; Imbrici et al. 2016). Voltage-gated sodium channels (Na_v_), calcium channels (Ca_v_), and potassium channels (K_v_) function by selectively conducting ions in response to membrane depolarization (Yu and Catterall 2004). A greater variety of stimuli including changes in temperature, pH, and mechanical pressure initiates gating in transient receptor potential (TRP) channels (Nilius and Owsianik 2011).

The ion conducting pathway of 6TM channels is centrally located at the interface between the four domains (DI–DIV) of single-chain channels (Na_v_ and Ca_v_ from eukaryotes) or between the four subunits of tetrameric channels (TRP, K_v_, and prokaryotic Na_v_s). The transmembrane helices S5 and S6 of each domain or subunit form the pore module and helices S1–S4 form the voltage-sensor in voltage-gated channels or auxiliary subunits in TRP channels (Long et al. 2005a; Payandeh et al. 2011; Liao et al. 2013). Despite the shared transmembrane architecture, there is a distinct lack of sequence conservation within the 6TM family, reflecting the diversity of their functions. For example, sequence differences in the S5–S6 extracellular linkers (‘P-loops’) determine ion selectivity of the channel. Other extracellular linkers and the cytoplasmic domains vary significantly in sequence and length and comprise sites of glycosylation, tetramerization domains, sites of pore-occluding inactivation particles, and receptor sites for auxiliary cytoplasmic subunits, e.g., K_v_ channel β-subunits (Hoshi et al. 1990; West et al. 1992; Kreusch et al. 1998; Gulbis et al. 2000; Cronin et al. 2005; Pongs and Schwarz 2010; Powl et al. 2010). In the voltage-gated 6TM channels, the S4 section bares a conserved series of positively charged residues that are involved in sensing and relaying changes in the transmembrane potential into gating-related conformational rearrangements. This process involves propagation of the voltage sensor movement to the helical S4–S5 linker segments, which contact the pore-lining S6 helices and exert control over their conformation, thus regulating pore gating (Long et al. 2005b).

**Fig. 1:**
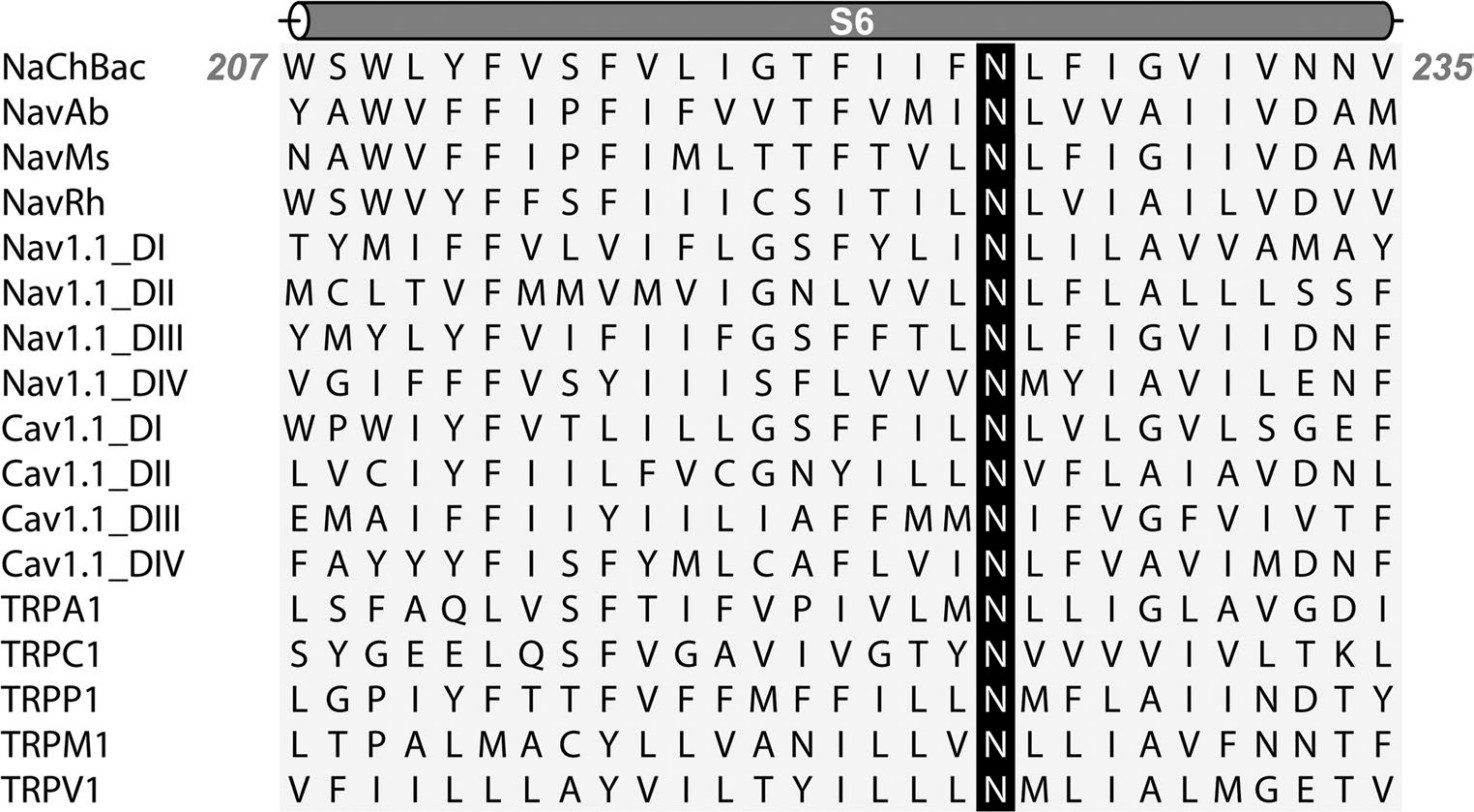
Sequence alignment of S6 segments. NaChBac, NavAb, NavMs, and NavRh are bacterial sodium channels and Nav1.1, Cav1.1 and TRP are human channels. The conserved S6 asparagine is shaded in* black*. Residue *numbers* are listed for the predicted start and end of the NaChBac S6 segment (depicted as a *cartoon cylinder* over the sequence). The sequence alignment was performed using T-COFFEE (http://www.ebi.ac.uk/tools/msa/tcoffee/)

Sequence alignment of a representative selection of 6TM channels (Fig. 1) demonstrates that the diversity of 6TM channel primary sequences extends to the pore-lining S6 helices. A remarkable exception to this variance is an asparagine residue (N225 in NaChBac) located near the center of each S6 helix in Na_v_, Ca_v_, and TRP channels (although completely absent from K_v_ channels). This conservation suggests that it may play an important role in channel function and pharmacology (Nau et al. 1999; McNulty et al. 2006). Supporting evidence for this hypothesis is the numerous and diverse channelopathies associated with mutation of this asparagine: long QT and Brugada syndrome in the cardiac channel Nav1.5 (Xiao et al. 2001; Itoh et al. 2005; Zimmer and Surber 2008), the pain syndrome hereditary erythromelalgia in the peripheral neuronal Nav1.7 (Sheets et al. 2007), paramyotonia congenita in the skeletal muscle Nav1.4 isotype (Lehmann-Horn et al. 2011) and severe myoclonic epilepsy in infancy in the neuronal Nav1.1 (Sugawara et al. 2003).

The study of 6TM channels has been aided by the discovery of bacterial homo-tetrameric Na_v_s that, in contrast to their larger, more complex and single-chained eukaryotic Na_v_ counterparts, can be over-expressed and purified in milligram quantities for structural studies. NaChBac from* Bacillus halodurans* was the first member to be identified and characterized electrophysiologically (Ren et al. 2001), biochemically (Nurani et al. 2008), and structurally using the highly sensitive technique of synchrotron radiation circular dichroism (SRCD) (Powl et al. 2010). Recent high-resolution crystal structures of the NaChBac homologues NavAb from *Arcobacter butzleri* (Payandeh et al. 2011), NavRh from *Rickettsiales* sp. *HIMB114* (Zhang et al. 2012) and NavMs from *Magnetococcus* sp. (McCusker et al. 2012) have provided structural insight into ion selectivity, gating, and inactivation mechanisms (Payandeh et al. 2012).

We employed the NaChBac channel as a model system to investigate the role of the conserved S6 asparagine. Our hypothesis was that the elimination of this asparagine through mutagenesis would produce a channel with modified biophysical properties. Of the numerous mutants generated, only N225D expressed functional channels. We compared the structural and functional properties of wild-type (WT) NaChBac with this N225D mutant and also analyzed high-resolution structures of homologous channels to gain insight into the structural consequences of replacing this S6 asparagine with aspartate.

## Materials and methods

### Site-directed mutagenesis and expression

NaChBac cDNA in the vector pTracer-CMV2 was obtained from Dr. Dejian Ren (Lab of Dr. David E. Clapham, Howard Hughes Medical Institute, Children’s Hospital, Harvard Medical School, Boston, MA, USA). The channel gene was sub-cloned into the pET15b vector as described (Nurani et al. 2008) for bacterial expression with an added N-terminal hexa-histidine affinity tag. Single-point mutations of the NaChBac N225 position were introduced into pTracer-CMV2 plasmid using the Quikchange protocol (Stratagene). The N225D single-point mutation was introduced into both the pTracer-CMV2 and pET15b plasmids using 5′-tgtcttaatcggtacgtttatcatctttgacttgtttatcggtgtaa-3′ and 5′-ttacaccgataaacaagtcaaagatgataaacgtaccgattaagaca-3′ primers. Following digestion of original DNA template with DpnI (Fermentas), 2 μl of PCR reaction was used to transformed DH5α *Escherichia coli* cells (New England Biolabs) with selection for resistance to ampicillin (100 μg/ml). The N225D mutations of both plasmids were confirmed by DNA sequencing.

WT NaChBac and the N225D mutant were over-expressed in C41 (DE3) *E. coli* cells and purified to homogeneity by immobilized-nickel affinity chromatography as described (Powl et al. 2010). Size-exclusion chromatography using a Superdex 200 10∕300 (GE Healthcare) column provided the final purification step and channel proteins were eluted in a buffer of 20 mM sodium phosphate; pH 7.8, 0.3% Cymal-5, 50 mM NaCl. Samples were concentrated using a Vivaspin (Sartorius) with a 50-kDa molecular weight cut-off.

### Synchrotron radiation circular dichroism (SRCD) spectroscopy

SRCD spectra were collected on beamline CD1 at the ISA synchrotron, University of Aarhus, Denmark. Thermal denaturation experiments were conducted using a protein concentration of ~2.5 mg/ml, which was determined immediately prior to data collection from the absorbance at 280 nm measured on a NanoDrop 1000 UV/Vis spectrophotometer. Protein samples were loaded into a 0.0024-cm pathlength quartz Suprasil demountable cell (Hellma UK), and spectra measured in the wavelength range of 260–175 nm with a 1 nm step size and a dwell time of 2 s. Three replicate spectra were measured at each calibrated temperature between 20 and 85 °C. The temperature was raised in 5 °C steps, allowing 3 min for equilibration at each temperature. The first and last spectrum collected at each temperature were compared to confirm that the sample had reached equilibrium prior to the measurements being made.

The replicate spectra were averaged and the average base line (buffer) spectrum was subtracted from each of the averaged sample spectra, which were then calibrated to a spectrum of camphorsulphonic acid obtained at the beginning of the beam-fill (Miles et al. 2004) and scaled to units of delta epsilon using a mean residue weight value of 114.35. All processing was carried out using CDTool software (Lees et al. 2004). Melt curves were derived from the CD signal at 193 nm and singular value decomposition carried out using the Sel2 algorithm within CDTool. Where appropriate, data were fitted with a Boltzmann function in Excel. Secondary structure analyses were carried out using the DichroWeb online server (Whitmore and Wallace 2008). The results from the CONTINLL, SELCON3 and CDSSTR algorithms (Sreerama and Woody 2000) using the SMP180 dataset (Abdul-Gader et al. 2011) were averaged and reported as ±1 standard deviation between the values obtained from the different algorithms. SRCD spectra have been deposited in the Protein Circular Dichroism Data Bank (PCDDB) (Whitmore et al. 2011) located at http://pcddb.cryst.bbk.ac.uk with the codes: wild type; CD0004079000-13, N225D; CD0004080000-13.

### Patch-clamp recordings and data analysis

HEK293t cells were grown in high-glucose Dulbecco’s modified Eagle’s medium (DMEM) supplemented with fetal bovine serum (10%), HEPES buffer (30 mM), penicillin (100 U/ml), streptomycin (100 µg/ml) (all Invitrogen) and taurine (1%) (Sigma–Aldrich). Cells were incubated at 37 °C with 5% CO_2_. HEK293t cells were transiently transfected with plasmids of NaChBac WT or NaChBac-N225D along with a reporter plasmid (CD8-pih3m) using the Nanofectin transfection reagent (PAA Laboratories; Pasching, Austria). After incubation for 12–15 h the cells were replated and used for experiments within 2–3 days. Transfection-positive cells were identified by immunobeads (anti-CD8 Dynabeads; Dynal Biotech, Invitrogen).

Whole-cell voltage clamp recordings were acquired with an Axopatch 200B amplifier (Molecular Devices, Sunnyvale, CA, USA). Currents were filtered at 5 kHz and sampled at 20 or 50 kHz using the pClamp10 software (Molecular Devices). Patch pipettes were pulled from borosilicate glass tubes (TW150-3, World Precision Instruments, Sarasota, FL, USA) and had a resistance of 1.5–2.5 MΩ. The external solution contained (in mM): 65 NaCl, 85 choline chloride, 2 CaCl_2_ and 10 HEPES [pH 7.4 adjusted with tetramethylammonium hydroxide (TEA-OH)]. The internal solution contained (in mM): 100 NaF, 30 NaCl, 10 EGTA and 10 HEPES (pH 7.2 adjusted with CsOH). In order to improve voltage-clamp conditions for NaChBac which produces large currents, we used an inverted sodium gradient to reduce the size of the sodium currents at the channel’s activation threshold (Cota and Armstrong 1989). We used the amplifier circuitry to compensate for 70% of the series resistance and to reduce the capacitance artifacts. Residual linear leak and capacitance artifacts were subtracted by using a P/4 protocol before the test pulse. Cells were held at a holding potential of −140 mV, pulse intervals were set to 40 s to allow for recovery from inactivation and to avoid run-down. All recordings were performed at room temperature.

**Fig. 2:**
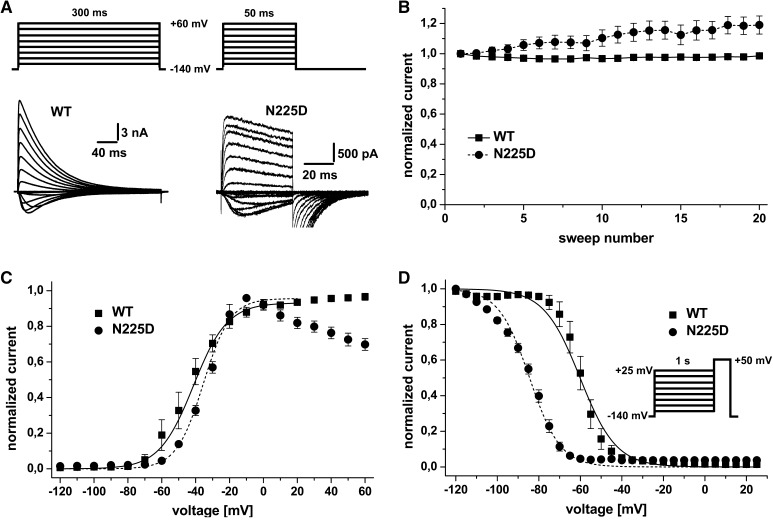
Analysis of activation and inactivation kinetics of NaChBac WT and mutant**. a** Representative current traces (*lower panel*) for activation of NaChBac-WT (*left*) and N225D mutant (*right*) expressed in HEK293t cells in response to the voltage protocol shown (*top panel*). Pulses for eliciting currents of N225D were shorter in order to prevent rundown. **b** Normalized current evoked by a series of test pulses to +50 mV at 40-s intervals for NaChBac-WT (300-ms test pulse length, squares, *n* = 7) and N225D mutant (50-ms test pulse length, circles, *n* = 10). No apparent run down is obvious. **c** Conductance-voltage relation of NaChBac-WT (*squares*) and N225D mutant (*circles*). The conductance was calculated from peak currents evoked by the protocol shown in **a**, normalized and plotted against the test pulse voltage. Data were fitted to a Boltzmann equation (*solid line* for WT, *dotted line* for N225D). **d** Voltage-dependence of steady-state inactivation of NaChBac-WT (*squares*) and N225D mutant (*circles*). Values were obtained by a test pulse to +50 mV following a conditioning prepulse incrementing in 5-mV steps (see *inset*). Peak currents were normalized to the peak current at the test pulse and plotted against the conditioning prepulse voltage

For activation, currents were evoked by 300 ms (wild type) or 50 ms (mutant) depolarizing test pulses ranging from −120 mV to +60 mV in 10 mV steps. As N225D showed some significant rundown when probed with normal 300-ms pulses, we reduced the pulse to 50 ms. This prevented rundown and the slight potentiation observed in Fig. 2 was small enough not to affect our results to a larger extent. Conductance *G* was calculated using the following equation: *G* = *I*_Na_/(*E*_m_ − *E*_rev_), where *I*_Na_ is the peak current, *E*_m_ is the corresponding voltage and *E*_rev_ is the estimated reversal potential. The reversal potential was extrapolated by fitting IV curves and was estimated to be between −15 and −20 mV. Normalized voltage-conductance curves were fitted to a Boltzmann equation:

$$ G/G_{\hbox{max} } = 1/\left( {1 + \exp \left[ {\left( {V - V_{1/2} } \right)/kv} \right]} \right) $$

where *G* is the conductance, *kv* is the the slope factor, *V*_1/2_ is the potential of half maximal activation, *G*_max_ is the maximal conductance. Current densities were calculated by dividing the maximal inward current in pA by the cell capacitance in pF as read from the amplifier.

For inactivation, currents were evoked by a test pulse to +50 mV following a 1-s prepulse ranging from −120 mV to +25 mV in 5-mV steps, normalized to the current measured following a prepulse to −120 mV and plotted against the conditioning prepulse potential. Length of the test pulse was 300 ms for WT and 50 ms for mutants. Normalized current–voltage curves were fitted to a Boltzmann equation (see above). All data are shown as mean ± SEM.

Origin 8.1G software (OriginLab Corp., Northampton, MA, USA) was used to perform additional curve fitting and analysis. An unpaired Student’s *t* test was used to test for statistical significance. A *p* value <0.05 was considered significant.

### Structural analysis of 6TM channels

The following coordinate files of 6TM channels with a S6 asparagine and a ≤3.5-Å resolution (where side chains should be resolved) were retrieved from the Protein Data Bank (Berman et al. 2000): PDB IDs 3J5P, 3RVY, 3RVZ, 3RW0, 3ZJZ, 4CBC, 4DXW, 4EKW, 4F4L, 4LTO, 4MS2, 4MTF, 4MTG, 4MTO, 4MVM, 4MVO, 4MVQ, 4MVR, 4MVS, 4MVU, 4MVZ, 4MW3, 4MW8, 4OXS, 4P2Z, 4P30, 4P9O, 4P9P, 4PA3, 4PA4, 4PA6, 4PA7, 4PA9, 4X88, 5BZB, 5EK0, 5IRZ, and 5IWK. The ‘Compute H-bonds’ function of Deepview software (Guex et al. 1999) was used to detect hydrogen bonds in each channel structure. Figures were produced using PyMOL (DeLano Scientific, San Carlos, CA, USA).

## Results

### N225D mutation of NaChBac shifts steady-state inactivation to more hyperpolarized potentials

We generated aspartate, leucine, alanine, threonine, histidine, and lysine mutations of the NaChBac N225 residue. The function of each mutant was assessed using the whole-cell patch-clamp technique but only the N225D mutant was found to produce currents.

Both WT and the N225D mutant expressed well in HEK293t cells and displayed voltage-dependent activation and inactivation (Fig. 2a). The current density of the mutation was lower compared to WT, probably due to an enhanced inactivation (see below, current densities: 879.48 ± 186.74 pA/pF, *n* = 8 for WT and 130.53 ± 15.96 pA/pF, *n* = 10 for N225D; *p* < 0.001, unpaired *t* test). In order to assure stable recording conditions, we chose a holding potential of −140 mV and a test pulse length of 300 ms for WT and 50 ms for the N225D mutant, as the latter was very sensitive to run-down. As shown in Fig. 2b, both WT and the N225D mutation did not display any current loss during a prolonged period of repetitive stimulation to +50 mV (300-ms pulse length). The results showed that even a slight run-up for the recording condition chosen for the N225D mutation would, if anything, counteract a current loss. Therefore a run-down of the channels under our experimental conditions is unlikely to interfere with the results.

The voltage dependence of activation was unchanged in the mutant channel compared to WT. Voltages for half maximal activation did not differ significantly between WT (−40.94 ± 4.15 mV, *n* = 10) and mutant (−33.29 ± 0.55 mV, *n* = 10, *p* = 0.095, unpaired *t* test), and also the slope factor did not change (Fig. 2c; WT: 10.18 ± 1.08; N225D: 8.39 ± 1.84). Interestingly, the conductance of mutant channels showed a marked decay at higher voltages, which suggests that inactivation affects the channels at these positive potentials. As we have shown in Fig. 2b, with our experimental settings, run-down is very unlikely to be the reason for this current decline. NaChBac inactivation is voltage-dependent and more pronounced at higher potentials (Fig. 2a). The mutant has an enhanced inactivation compared to WT and it is therefore likely that the channels begin to inactivate before the current reaches its peak. This could account for the deviation of the conductance curves at higher potentials (Fig. 2c).

**Fig. 3:**
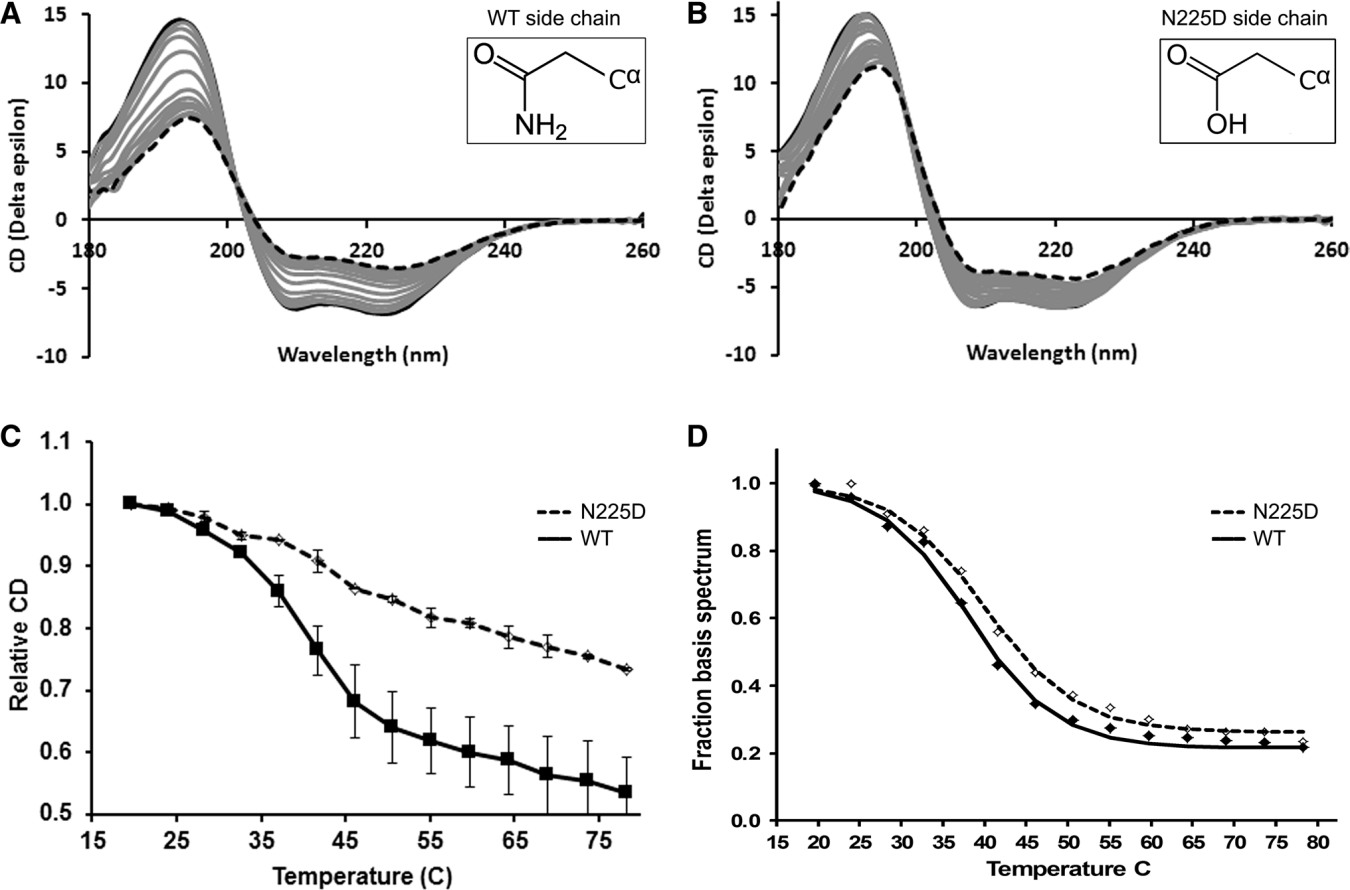
Thermal denaturation studies of NaChBac using SRCD spectroscopy. SRCD spectra of **a** WT and **b** N225D NaChBac measured over the temperature range of 20 °C (*black solid lines*) to 85 °C (*black dotted lines*) in 5 °C increments. *Grey* spectra are intermediate temperatures. *Insets* show the chemical structure of each 225 residue side chain. **c** Thermal denaturation curves of WT (*solid line*) and N225D (*dashed line*) derived from plotting the signals at 193 nm of spectra (**a**, **b**) versus temperature. *Error bars* represent 1 standard deviation between two repeats. **d** Fraction of first basis spectra as a function of temperature obtained by carrying out singular value deconvolution analyses (32) on the full spectra. The data are fitted to a Boltzmann function

In contrast to activation, steady-state inactivation properties of the mutant channel displayed a pronounced hyperpolarizing shift of *V*_1/2_ by 24.7 mV (Fig. 2d; WT: −59.9 ± 2.24 mV; N225D: −84.6 ± 1.09 mV, *p* < 0.001). The slope factor for the N225D mutant is almost doubled (*p* < 0.001; WT: 4.08 ± 0.29; N225D: 8.55 ± 0.30) compared to WT, hence the curve is less steep. This enhanced inactivation of the mutant channel might be the cause of the run-down that we found when using longer pulses.

### N225D mutation renders NaChBac protein more thermostable

The secondary structure composition and thermostability of WT and N225D NaChBac were determined using SRCD spectroscopy. In comparison with conventional CD spectroscopy, the use of synchrotron radiation as the light source for collecting UV CD measurements provides a significant improvement in the signal-to-noise ratio, especially at low wavelengths (around the ~190 nm peak). Our previous use of SRCD with NaChBac revealed the helical nature of its C-terminal tetramerization domain (Powl et al. 2010), the relative contribution that the transmembrane and extramembraneous domains make to channel thermostability (Powl et al. 2012) and, of particular relevance for the current study, allowed detection of a structural change in the pore arising from mutation of the putative gating-hinge position (G219) of the S6 helix (O’Reilly et al. 2008).

Both WT and mutant NaChBac channels have essentially identical SRCD spectra at 20 °C (Fig. 3a, b), with negative peaks at 222 and 209 nm and a large positive peak at ~193 nm indicative of a folded protein. Analysis of the SRCD data (Table 1) confirms a comparable helicity of 64–66% for both proteins at this temperature, matching previous secondary structure determinations of NaChBac channels solubilized in either Cymal-5 or dodecyl maltoside detergents (Nurani et al. 2008, 2010; Powl et al. 2012). The helical structure is primarily due to the residues in the transmembrane helices and the C-terminal cytoplasmic domain.

**Table 1:** Secondary structure analysis based on SRCD data

	Helix (%)	Sheet (%)	Disorder (%)	Tm
WT (20 °C)	64 ± 2	5 ± 1	21 ± 2	38 °C
WT (35 °C)	61 ± 1	5 ± 1	21 ± 2	
WT (85 °C)	37 ± 3	21 ± 1	32 ± 2	
N225D (20 °C)	66 ± 2	5 ± 2	19 ± 1	40 °C
N225D (35 °C)	62 ± 1	6 ± 2	22 ± 2	
N225D (85 °C)	51 ± 3	13 ± 1	26 ± 3	

Tm values are calculated from the curves in Fig. 3c. The ± values indicate one standard deviation between values calculated by different algorithms

The entire SRCD spectrum was monitored during thermal denaturation studies to determine if a difference between the WT and mutant proteins could be discerned. Relative to the N225D mutant, the spectra of the WT channel underwent greater changes in shape and magnitude with increasing temperature (Fig. 3). The maximal changes were observed at 193 nm (Fig. 3c, d), a wavelength that reports on the decrease in helix content and increase in disordered or ‘other’ structures and therefore protein denaturation. At the maximum temperature of 85 °C, the mutant retains a helical content of 51 ± 3% whereas with the WT channel helicity is reduced to 37 ± 3%.

Singular value decomposition was used to decompose the spectral data comprising the melt into principal components present. The number of principal components identified indicates the number of spectral types in each spectrum, which in this case is two: one representing the folded protein, the other a disordered structure. The plot in Fig. 3d shows the fraction of the first (folded) component present in the sample as a function of temperature and the S-shaped curve indicates a cooperative unfolding is observed with both WT and mutant.

Together, these results demonstrate that at low and physiological-relevant temperatures, the WT and mutant channels have very similar secondary structures, indicating that N225D represents a sterically conservative substitution that does not disrupt folding or introduce a gross distortion to the channel structure. The mutant channel is, however, more thermostable than the WT. The resistance of NaChBac-N225D to unfolding may reflect a change in channel flexibility that produces a shift in the equilibrium of gating-related conformational states (see “Discussion”).

### Polar interactions in 6TM channel structures

**Fig. 4:**
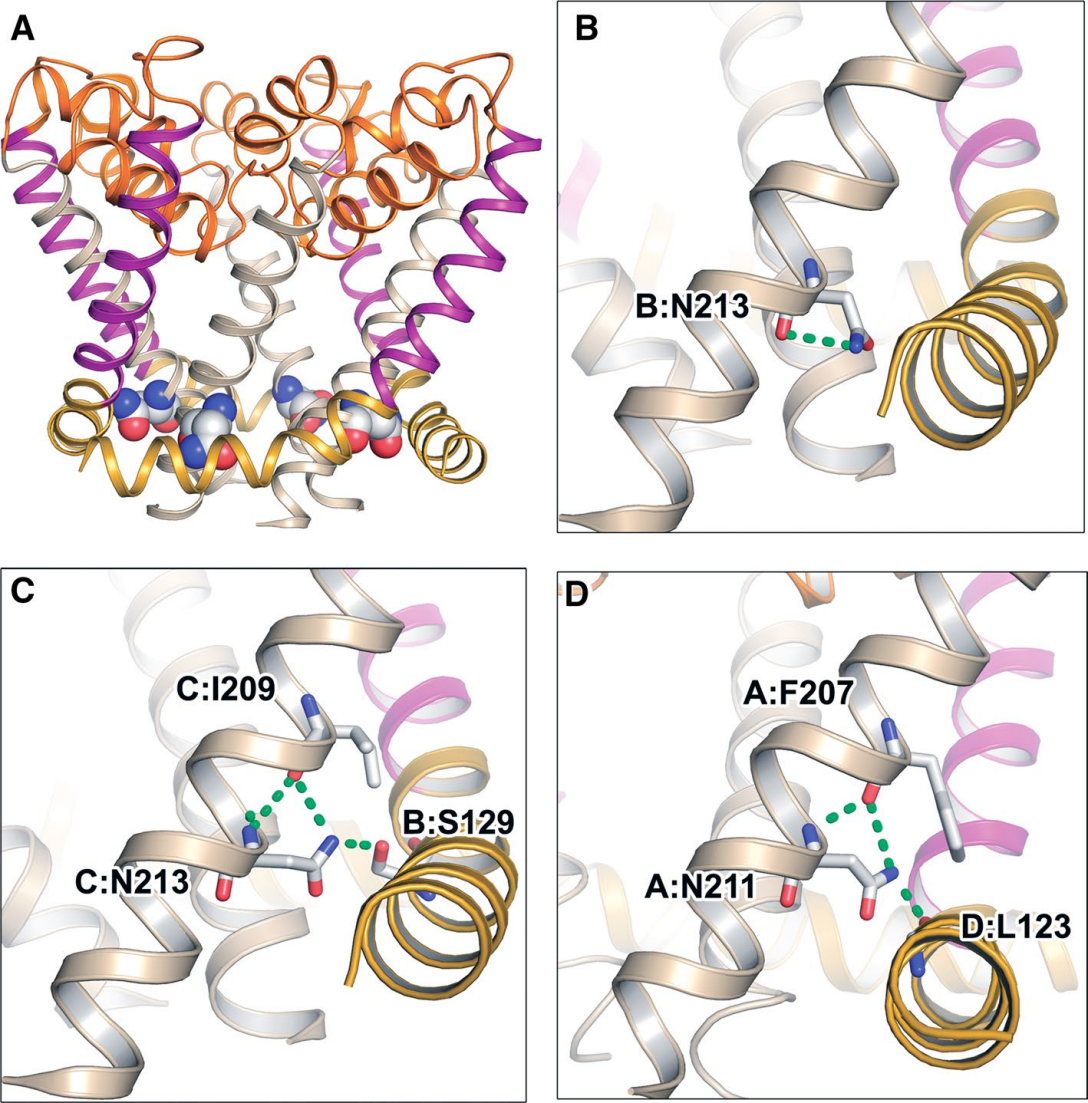
Hydrogen bonds involving S6 asparagines in the crystal structures of NavRh (PDB ID 4DXW) and NavAb (PDB ID 4EKW) in inactivated conformations. **a** Transmembrane view of the NavRh pore module shown in *ribbon* format with S4-S5 linkers *colored yellow*, S5 helices *purple*, P-loops *orange*, S6 helices *beige*. The conserved S6 asparagines are in* spacefill*. **b–d** Asparagines are shown as* sticks* and hydrogen bonds as *green dashes* for **b** NavRh chain B N213, **c** NavRh chain C N213, and **d** NavAB chain A N211

All available high-resolution structures of members of the 6TM channel family were examined to identify hydrogen bonds involving the S6 asparagine. The side chain of this conserved residue was found to engage in three different hydrogen bond configurations in the NavRh and NavAb channels crystallized in putatively inactivated states (Payandeh et al. 2012; Zhang et al. 2012) (Fig. 4). Both of these channels are characterized by asymmetric structures where the selectivity filters are apparently deformed (and proposed to be non-conducting) and two S6 helices on opposite sides of the pore are in closer contact at the pore cytoplasmic constriction than the second pair of S6 helices.

Figure 4b shows that in chain B (PDB ID 4DXW) of the NavRh channel the S6 N213 side chain donates a hydrogen bond to its own backbone carbonyl group. In contrast, in chain C the N213 side-chain amine group is positioned to donate two hydrogen bonds simultaneously (Fig. 4c). The first is to the hydroxyl group of a serine (S129) located on the S4–S5 linker of the adjacent chain. The second hydrogen bond is to the backbone carbonyl group of a residue (I209) located on the same S6 helix. A similar hydrogen bond network is found in the NavAb structure (PDB ID 4EKW) as the amine group of the S6 asparagine (N211) is positioned to form a hydrogen bond with a backbone carbonyl of a preceding residue (F207) on the S6 helix (Fig. 4d). There is also a simultaneous hydrogen bond to the S4–S5 linker of the adjacent chain but, in contrast to the NavRh structure, the acceptor group is a backbone carbonyl group (of residue L123) instead of a side-chain group.

## Discussion

We mutated the NaChBac sodium channel to explore the structural and functional role of N225, which represents the only residue that is conserved throughout the entirety of the Na_v_, Ca_v_, and TRP sequences (Fig. 1). The effect of the N225D mutation was to increase the thermostability of the channel and to produce a −25 mV shift in the *V*_1/2_ of inactivation.

In the absence of a transmembrane potential, purified and detergent-solubilized NaChBac channels are predicted to be in equilibrium between the closed, activated, and inactivated conformations. Conditions that produce a shift in this equilibrium, such as the binding of drugs and toxins or the effects of mutagenesis or truncations, have been monitored using CD studies (Cronin et al. 2003, 2005; Nurani et al. 2008; O’Reilly et al. 2008; Powl et al. 2010, 2012). Previously we reported that the G219S mutation of NaChBac produces a more thermally stable channel (O’Reilly et al. 2008). We rationalized that a substitution at this putative gating-hinge position produces a reduced S6 flexibility, consequently shifting the conformational equilibrium towards the closed state. Analysis of homology models showed that there is a 25% increase in van der Waals contact when the splayed S6 helices of the open-state channel are brought into closer contact in the closed state (O’Reilly et al. 2008). We proposed that increased inter-monomer contacts stabilize the channel structure and thereby contribute to thermal resilience. The effects of N225D closely resemble those of G219S, where no apparent differences in secondary structure are present between WT and mutant at lower temperatures but a resistance to thermal denaturation and significant retention of helical content are found at higher temperatures. Therefore, in analogy to G219S, our results suggest that the N225D mutation shifts the equilibrium to conformations where greater stabilizing inter-domain contacts are present. Structures of NavRh (Fig. 4a) and NavAb homologues of NaChBac captured in inactivated conformations (Payandeh et al. 2012; Zhang et al. 2012) show that S6 helices are closely apposed in the inactivated state. Therefore, the greater thermostability of the N225D mutant may be due to channels stabilized in their inactivated state (see “Discussion” below). Our electrophysiology characterization of the N225D mutant indeed shows that there is a major (−25 mV) shift in the voltage dependence of inactivation, making this functional state more likely to be adopted in comparison to the WT channel.

Many N225 mutations were generated to investigate how a range of physicochemical changes to the 225 side chain would affect channel activity. However, only the N225D mutation was found to produce functional channels. Our findings are consistent with reports of N225A and N225P mutagenesis of NaChBac that also produced non-functional channels (Zhao et al. 2004; Lee et al. 2012). The side chain of the conserved asparagine is located between a S6 helix and S4–S5 linker (Fig. 4a). As the coordinated movement of these channel elements is critical for the coupling of voltage-sensing with pore gating (Long et al. 2005b), variations in side-chain size at the asparagine position may alter spacing between the two helices and disrupt channel activity. This may explain why only the sterically conservative N225D mutation (see insets in Fig. 3a, b) produce functional channels.

Analysis of current high-resolution 6TM channel structures shows that hydrogen bonds involving the S6 asparagine side chain feature predominantly in the putatively inactivated sodium channels NavRh and NavAb. In two chains of each channel, the asparagine side-chain amine donates an inter-monomer hydrogen bond to a S4–S5 linker, which was previously proposed to be important for the channel inactivation process (Payandeh et al. 2012). Our findings support this hypothesis as the N225D mutation in NaChBac substitutes the side chain amine with a carboxylate group—eliminating its ability to donate a hydrogen bond to the S4–S5 linker—and the functional outcome is modified channel inactivation. In NavRh, the N213 side-chain amine also donates intra-subunit hydrogen bonds to backbone carbonyl groups in different conformations of the S6 helices (Fig. 4b, c). One intriguing possibility is that these hydrogen bonds play a role in stabilizing functionally relevant conformations of the S6 helices and, when abolished in NaChBac through N225D mutagenesis, the channel enters an inactivated state more readily. Conformational changes of the S6 helices may be propagated to the P-loop regions (Fig. 4a) where structural rearrangement of the selectivity filter to a non-conducting state produces C-type inactivation (Payandeh et al. 2012).

In eukaryotic Na_v_ channels, the mutation of the conserved S6 asparagine induces a wide range of effects that vary depending on the Na_v_ isotype, domain, and the nature of the substitution. For example, the N406D mutation in domain I of Na_v_1.5 induces a shift of activation to more positive potentials, while fast inactivation remains unaltered (McNulty et al. 2006). The corresponding mutation (N434D) in Na_v_1.4 depolarizes voltage dependence of fast and slow inactivation (Nau et al. 1999) while the domain III N1466D mutation in Na_v_1.2 results in almost no change in activation or fast inactivation but almost completely abolishes slow inactivation (Chen et al. 2006). This variation in effect is found with other asparagine mutations (Wang et al. 1998; Nau et al. 2000; Yarov-Yarovoy et al. 2001; Wright 2001; Xiao et al. 2001; Yarov-Yarovoy et al. 2002; Sheets et al. 2007; Browne et al. 2009) and reflects the underlying complexity of these pseudo-tetrameric channels, where each domain displays individual activation and inactivation kinetics (Chanda and Bezanilla 2002; Chanda et al. 2004). In addition, the lack of equivalent structural elements in NaChBac such as the DIII-DIV linker, which forms the fast inactivation particle in eukaryotic Na_v_ channels (West et al. 1992), prevents us from fully generalizing our findings using the NaChBac model system.

Our data provide evidence supporting an important functional role for the conserved S6 asparagine in NaChBac. We found that the 225 position is sensitive to substitutions as only the N225D mutation produced functional channels. The inability to form one or more hydrogen bonds in this mutant channel may account for its increased thermostability and enhanced inactivation. Finally, we suggest that the side-chain amine group of the conserved S6 asparagine may play an important functional role as a hydrogen bond donor in other 6TM channels.
